# The C-seal trial: colorectal anastomosis protected by a biodegradable drain fixed to the anastomosis by a circular stapler, a multi-center randomized controlled trial

**DOI:** 10.1186/1471-2482-12-23

**Published:** 2012-11-15

**Authors:** Ilsalien S Bakker, Annelien N Morks, Henk O ten Cate Hoedemaker, Johannes G M Burgerhof, Henri G Leuvenink, Rutger J Ploeg, Klaas Havenga

**Affiliations:** 1 Department of Surgery, University Medical Center Groningen, Groningen, the Netherlands; 2 Department of Surgery, Medical Center Leeuwarden, Leeuwarden, the Netherlands; 3 Department of Epidemiology, University Medical Center Groningen, Groningen, the Netherlands; 4 Nuffield Department of Surgical Sciences, University of Oxford, Oxford, United Kingdom

## Abstract

**Background:**

Anastomotic leakage is a major complication in colorectal surgery and with an incidence of 11% the most common cause of morbidity and mortality. In order to reduce the incidence of anastomotic leakage the C-seal is developed. This intraluminal biodegradable drain is stapled to the anastomosis with a circular stapler and prevents extravasation of intracolonic content in case of an anastomotic dehiscence.

The aim of this study is to evaluate the efficacy of the C-seal in reducing anastomotic leakage in stapled colorectal anastomoses, as assessed by anastomotic leakage leading to invasive treatment within 30 days postoperative.

**Methods:**

The C-seal trial is a prospective multi-center randomized controlled trial with primary endpoint, anastomotic leakage leading to re-intervention within 30 days after operation. In this trial 616 patients will be randomized to the C-seal or control group (1:1), stratified by center, anastomotic height (proximal or distal of peritoneal reflection) and the intention to create a temporary deviating ostomy. Interim analyses are planned after 50% and 75% of patient inclusion. Eligible patients are at least 18 years of age, have any colorectal disease requiring a colorectal anastomosis to be made with a circular stapler in an elective setting, with an ASA-classification < 4. Oral mechanical bowel preparation is mandatory and patients with signs of peritonitis are excluded. The C-seal student team will perform the randomization procedure, supports the operating surgeon during the C-seal application and achieves the monitoring of the trial. Patients are followed for one year after randomization en will be analyzed on an intention to treat basis.

**Discussion:**

This Randomized Clinical trial is designed to evaluate the effectiveness of the C-seal in preventing clinical anastomotic leakage.

**Trial registration:**

NTR3080

## Background

Anastomotic Leakage (AL) is the most important complication after colorectal surgery and leads to high rates of morbidity, prolonged hospitalization, commonly requires re-interventions, and can result in death [1].

The incidence of AL varies in the literature between 4 and 20% [2-6]. This considerable variation may be due to the fact that different definitions are used. The International Study Group of Rectal Cancer (ISGRC) proposed a standardized definition for AL according to clinical grading [7]. AL is defined as a defect of the intestinal wall at the anastomotic site leading to a communication between the intra and extraluminal compartments. An abscess in the proximity of the anastomosis is considered to be AL. The severity of AL is classified in three grades. Grade A AL corresponds to the definition ‘radiologic leakage’ for which no intervention is required. Grade B AL requires an active therapeutic intervention but could be managed without relaparotomy. Grade C AL requires a relaparotomy [7].

Recent data from the Dutch Surgical Colorectal Audit (DSCA) showed an AL rate of 11% leading to re-intervention in the Netherlands [8].

In order to reduce the incidence of AL the C-seal is developed. The C-seal is an intraluminal biodegradable drain which is stapled to the anastomosis with a circular stapler [9]. The C-seal covers the intraluminal side of the anastomosis and prevents extravasation of intracolonic content to the peritoneal cavity in case of anastomotic dehiscence.

In previous studies performed in the University Medical Center Groningen (UMCG) and in some other clinics a total amount of 52 patients were treated with the C-seal. The pilot study of fifteen patients, who underwent a colorectal resection with a C-seal, showed that the application of the C-seal was feasible and there were no clinical or radiological signs of leakage [10]. Also the subsequent phase II study with 37 patients confirmed the feasibility of the C-seal by both surgeons and by patients. There were no serious adverse events reported related to the C-seal [11]. Furthermore this study showed promising results in the reduction of clinical AL, which led to the initiation of a randomized controlled trial, the C-seal trial. This trial aims to compare the outcome for stapled colorectal anastomoses made with and without a C-seal.

The main objective of the C-seal trial is to evaluate the efficacy of the C-seal in reducing AL in stapled colorectal anastomoses, as assessed by AL leading to invasive intervention within 30 days postoperative.

## Methods

### Design

The C-seal trial is a prospective multi-center randomized controlled trial, initiated by the department of surgery, division of abdominal surgery of the UMCG, the Netherlands. The study will be performed in approximately 35 clinics.

In this trial 616 patients will be randomized equally between the C-seal group and the control group. The study protocol is approved by the central medical ethics committee of the UMCG. In all participating centers a local feasibility agreement is arranged. Patients will be followed until one year after randomization.

### Participants

Eligible patients are diagnosed with any colorectal disease requiring a colorectal anastomosis in an elective setting, have a minimum age of 18 years and an ASA classification < 4. Preoperative mechanical bowel preparation is mandatory, but can be omitted if the patient has a previously created deviating ostomy that will remain in situ. Patients with clinical signs of peritonitis and patients who underwent major surgery 30 days prior to the operation or have major surgery planned within 30 days after the procedure, are excluded.

All eligible patients visiting the surgical outpatient department in participating centers will be informed about the trial. Informed Consent must be obtained before inclusion.

The participating study sites should comply with the recent guidelines of the Dutch Society of Surgery to perform colorectal surgery (Normering Chirurgische behandeling. NVvH January 2011). The operating surgeons are adequately trained in the application of the C-seal.

### Intervention

**Figure 1:**
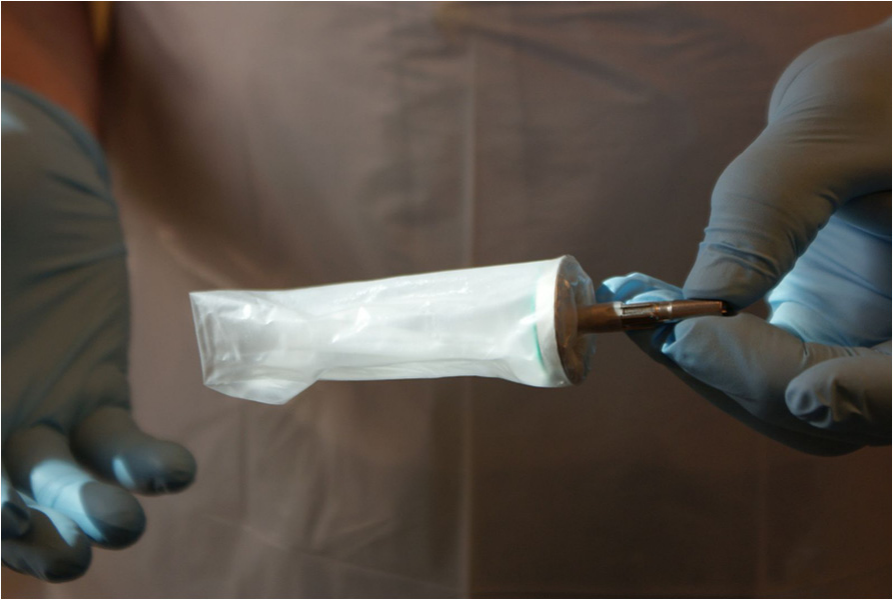
C-seal attached to the anvil of the circular stapler.

**Figure 2:**
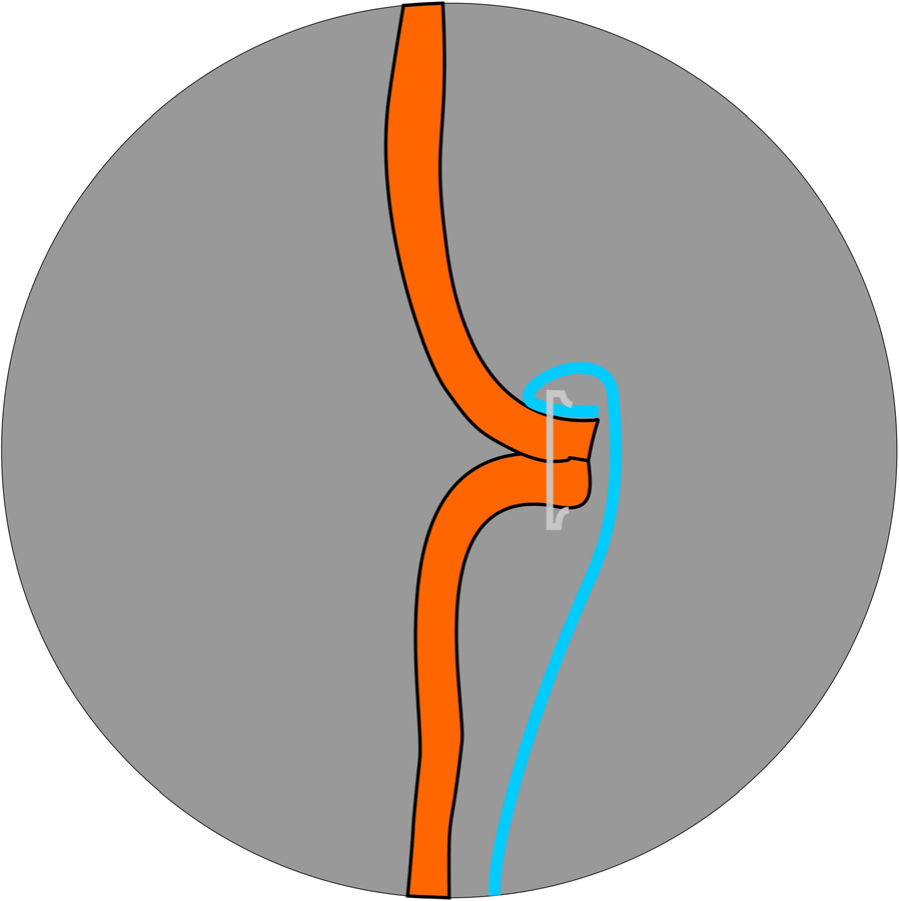
Intraluminal attachment of the C-seal after the staple procedure.

Fifty percent of the study population will be randomized to the C-seal group and 50% to the control group. The C-seal is stapled to the anastomosis with a circular stapler. Before creating the anastomosis the C-seal is attached to the anvil of the circular stapler (Figure 1) and together they are inserted in the proximal bowel loop. After firing the circular stapler the C-seal is stapled to the mucosal side of the afferent loop (Figure 2). When the stapler is removed from the anus, the C-seal is brought through the anus and cut from the stapler anvil. The application of the C-seal to the anvil is trained in a practicum session provided to the participating operating surgeons and local research group.

Postoperatively the C-seal will be in situ for approximately two weeks. The C-seal degrades in time and secretes through the anus together with bowel contents.

### Outcome

The primary endpoint of the C-seal trial is the incidence of AL leading to invasive treatment within 30 days after surgery. AL is defined based on the classification according the ISGRC classification.

Secondary endpoints are the number of dismantled anastomoses, the number of ostomies created, the number of ostomies present after 1 year, the total duration of hospital stay, the interval between primary surgery and the diagnosis of AL, the incidence of late AL (≥ 30 days and within 1 year) and a differentiation between grade A, B or C AL classified according the ISGRC [7].

### Sample size

The sample size of 616 patients is determined with a power analysis based on a power of 90%, a significance level of 5%, AL percentage of 4% in the C-seal group and 10% AL in the control group.

### Randomization

Patients are equally distributed to either the C-seal or the control group. In an attempt to overcome possible bias, the randomization procedure is performed in the operating room during surgery. The randomization procedure is performed by a C-seal student with an online randomization system. The randomization is stratified for center, high or low anastomosis (respectively proximal or distal of the peritoneal reflection) and the intention to create a temporary deviating ostomy.

### Statistical analysis

Primary and secondary endpoints will be analyzed on an intention to treat basis. The groups will be compared by means of a Fisher’s exact test.

After 50% and 75% of patient enrollment an interim analysis will be performed based on Snapinn’s stopping rule [12].

## Discussion

This study investigates the efficacy of the C-seal in the prevention of clinical AL in stapled colorectal anastomoses. The concept of the protection of a colorectal anastomosis by means of an intraluminal device has already been described [13-15]. Based on the idea that intraluminal devices in colorectal anastomoses protect the fecal stream from anastomotic contact, there will be no leakage if anastomotic dehiscence develops. Under the device a perforation can still arise, but because there is no fecal spill this probably will not lead to a fecal peritonitis. Local inflammation may cause adhesions and cover the defect and symptoms of a possible leak are probably less severe [13,16].

It is also hypothesized that AL in colorectal anastomoses is frequently caused by poor vascularization through intraoperative mechanical manipulation [17,18], often from the distal rectal stump [19]. Since the C-seal is attached to the proximal bowel loop, the C-seal remains in situ if the rectal stump is poorly vascularized and leads the fecal stream in the first postoperative weeks outside the body without contact with the rectal wall. After about two weeks the biodegradable drain degrades and is excreted through the anus.

In this study the required sample size is determined based on the current AL rate in the Netherlands. To give a good reflection of the colorectal surgery in the Netherlands and in order to approach this AL incidence, 35 different hospitals, ranging from small referral hospitals to academic centers, are participating in the trial. The participation of this large number of clinics creates possible challenges in protocol adherence. To standardize procedures all operating surgeons are trained by the C-seal research team from the UMCG and during the surgery a student from the C-seal team attends the procedure. To overcome a possible bias regarding the participation of many clinics the randomization is stratified for center.

In addition to the stratification for center, there is also stratified for the intention to create a deviating ostomy and for anastomotic height. The stratification for the intention to create a deviating ostomy is performed to prevent selection bias. The outcome of randomization (C-seal or not) may influence operating surgeons in their decision to create a deviating ostomy. Therefore, the intention to create a deviating ostomy must be documented before the patient is randomized at the OR.

A limitation of the study may be that we have chosen not to stratify for neoadjuvant therapy. Most patients with rectal cancer in the Netherlands are treated with neoadjuvant radiotherapy. From the literature it is concluded that short scheme radiotherapy is not a significant risk factor for the occurrence of AL [20,21]. Results of studies with long scheme chemo radiation however are inconsistent in the influence on the incidence of AL. Some studies describe a significant increase in the occurrence of AL [22,23], while results from other studies did not found this increase in the incidence of AL [24,25].

Because a large study population will be included in this trial, the patients will be proportionally allocated to both groups.

With the results of this randomized controlled trial we compare the incidence of AL leading to re-intervention in patients treated with a C-seal to patients treated without a C-seal.

Secondary to this prospective trial a few side studies will be performed. With the use of intraoperative collected data of anaesthesiological parameters as oxygenation, blood tension, temperature and the use of vasoconstrictive medication eg, we attempt to identify potential contributing factors for the occurrence of AL. Also intraoperative data of the technical aspects of the stapling process are noted in the CRF to evaluate the surgical technique used to create an anastomosis in the Netherlands and the possible relation this could have in causing AL. Adjacent to the clinical trial translational research will be conducted with blood draws and bowel samples of the proximal and distal donuts of the included patients, collected during the surgery. With these patient materials we aim to correlate molecular tissue characteristics as pro-inflammatory proteins, Matrix Metallo Proteinases and C-reactive protein with clinical outcome. In blood plasma, indicators of inflammation, bacterial translocation and intestinal damage will be determined and will be correlated with the molecular profile of the patients.

## Competing interests

The authors declare that they have no competing interests.

## Authors’ contributions

All authors contributed to the accomplishment of this manuscript. IB wrote the manuscript. IB and AM made substantial contributions to conception and design of the study protocol. KH wrote the study protocol and revised the manuscript. JB provided the statistical design of the study. HL participated in the design and coordination of the trial. HTCH and RP participated in the design of the study and revised the manuscript. All authors read and approved the manuscript.

## Funding

Funding for this study is provided by Polyganics Innovations B.V, Groningen, the Netherlands. Polyganics manufactures the C-seal, they have no role in the design of the trial and they are not in any way involved in collecting and analyzing data or interpreting of the trial results.

## Pre-publication history

The pre-publication history for this paper can be accessed here:

http://www.biomedcentral.com/1471-2482/12/23/prepub
